# Economic Evaluation of Conservation through Use of an *Araucaria angustifolia* Provenance and Progeny Test

**DOI:** 10.3390/plants13182580

**Published:** 2024-09-14

**Authors:** José Arimatéia Rabelo Machado, Miguel Luiz Menezes Freitas, Daniela Ivana Paiva, Bruno Marchetti de Souza, Valderês Aparecida De Sousa, Karina Martins, Edilson Batista Oliveira, Ananda Virginia De Aguiar

**Affiliations:** 1 Departamento de Tecnologia e Inovação, Instituto de Pesquisas Ambientais, Rua do Horto, 931, São Paulo 02377-000, SP, Brazil; 2 Programa de Pós-Graduação em Ecossistemas Agrícolas e Naturais, Universidade Federal de Santa Catarina, Rodovia Ulysses Gaboardi, Km 3, Curitibanos 89520-000, SC, Brazil; 3 Faculdade de Engenharia de Ilha Solteira, Universidade Estadual Paulista “Júlio de Mesquita Filho”, Avenida Brasil, 56, São Paulo 15385-000, SP, Brazil; 4 EMBRAPA Floresta, Empresa Brasileira de Pesquisa Agropecuária, Estrada da Ribeira, Km 111, Colombo 83411-000, PR, Brazilananda.aguiar@embrapa.br (A.V.D.A.); 5 Departamento de Biologia, Universidade Federal de São Carlos, Rodovia João Leme dos Santos, Km 110, Sorocaba 18052-780, SP, Brazil

**Keywords:** Paraná-pine, multiple uses, sustainable forestry, genetic conservation, forest economics

## Abstract

*Araucaria angustifolia* is a species known for its valuable wood and nuts, but it is threatened with extinction. The plantation of forests for genetic resource conservation is a complementary strategy designed to reduce the species’ genetic variability loss. This study aimed to evaluate the technical and economic viability of *A. angustifolia* for genetic conservation through use. The analyzed provenance and progeny trial was established in 1982 in Itapeva, Brazil. It was structured using a compact family blocks design with 110 open-pollinated progenies from five natural populations, three replicates, ten plants per subplot, and 3.0 m × 2.0 m spacing. After 33 years, the trial was evaluated for total height, diameter at breast height, wood volume, and survival. The variance components and genetic parameter estimates were performed using Restricted Maximum Likelihood/Best Linear Unbiased Prediction methods (REML/BLUP) methods with the Selegen software (version 2014). The production and management scenarios were obtained using the SisAraucaria software (version 2003). Sensitivity analysis and economic parameter estimates were obtained through various economic evaluation methods using the Planin software (version 1995). In general, the genetic parameters indicated that the population has enough variability for both conservation and breeding purposes, suggesting technical viability for the establishment of a seed orchard. The economic parameters indicated that the commercialization of wood and araucaria nuts proved to be more profitable than wood production by itself. In conclusion, araucaria genetic conservation through use is a technically and economically viable ex situ conservation strategy.

## 1. Introduction

Information on the economic viability of conservation and production programs focusing on non-conventional tree species is scarce in technical–scientific studies, especially when it deals with expanding the use of these species outside their natural area. Additionally, studies analyzing the economic value of forests under different production scenarios are lacking. Transforming an alternative species into an economically valuable resource requires significant financial investments over an extended period. This need for high costs and time limits the ability of many countries, especially developing and underdeveloped ones, to undertake these projects. As a result, opportunities to utilize these forest resources to meet human demands are reduced [1,2].

*Araucaria angustifolia* (Bert.) O. Ktze (Paraná pine, araucaria) can serve as a model for this type of study due to its ecological, social, and economic importance [3]. This is particularly significant, as the high economic value of its wood has led to the species being exploited for several decades. Additionally, new properties of its seeds are being discovered, increasing their popularity and use across various industries [4,5].

The Paraná pine is one of the most significant tree species in Brazilian flora. In the past, it covered an area of 200,000 km^2^ in the southern region of Brazil (Ombrophilous Mixed Forest in the Atlantic Forest domain), and, during the 1970s, the high-quality timber from this conifer became the country’s main export product [6]. The intense exploitation of araucaria has led to a dramatic reduction in its natural forests, shrinking the Ombrophilous Mixed Forest in Brazil to less than 3% of its original area [7], leaving only small forest remnants [8]. Currently, several restrictions have been imposed on exploring this biome, particularly to protect its main species, *A. angustifolia* [8]. Recently, the risk of violating environmental laws has increased the use of exotic pine species for wood production in Brazil, discouraging the planting of araucaria [3]. Another major concern for araucaria in situ conservation is the reduced effective population size of the remaining forest fragments. This reduction can increase inbreeding and the loss of alleles due to genetic drift, potentially leading to genetic bottlenecks in the future [9]. Additionally, the species’ deficit and complex reproductive cycle can make the natural regeneration of small populations unfeasible [10]. Furthermore, araucaria may be strongly affected by climate change, necessitating that conservation strategies also consider the species’ niche map [10,11].

To reduce the pressure on natural araucaria forests, it is imperative not only to establish protected areas that are managed to sustain local economic growth [12] but also to develop genetic breeding and conservation programs in collaboration with local farmers [8]. These productive forests could serve as viable conservation alternatives. Proper management of these forests can align with conservation objectives, potentially creating active germplasm banks to supply seeds for new productive forests, facilitate natural forest recovery [13], and establish breeding populations. However, the costs associated with isolating large areas and harvesting seeds are prohibitively high. Ex situ genetic conservation programs offer numerous advantages, particularly in reducing expenses [14]. These areas can more readily and appropriately provide seeds from selected genotypes that are tailored to specific goals [15]. Given its vulnerability to climate change, socio-economic pressures, legal constraints, and low natural reproduction rates, ex situ conservation remains a crucial complementary measure to safeguarding araucaria genetic diversity [16].

The production of araucaria seeds (hereafter, nuts) as an alternative or supplementary activity to logging serves as a successful incentive to prevent species extinction, addressing the urgent need to conserve its genetic resources. Conservation through use is essential for assessing and making viable the production of araucaria wood and nuts [11]. When farmers engage in araucaria cultivation focused on nut production, conservation through use practices can be established and sustained as long as interest persists [3]. This interest could be sustained, given that the wood is highly valuable, and the uses and properties of the seeds are increasingly being described [4,5,17], turning them into a raw material for the production of various products across different industries, such as pharmaceuticals, dietary and nutritional products, packaging, and insecticides. This method stands out as one of the most effective ex situ conservation approaches for conserving the genetic resources of tree species. However, it alone cannot guarantee a continuous seed supply for future breeding programs [18,19]. Therefore, the aim of this study is to economically evaluate an araucaria provenance and progeny trial based on genetic and economic parameters and to promote the sustainable use of araucaria wood and nuts, combining genetic conservation and use in the development of breeding programs for the species.

## 2. Results

### 2.1. Scenario Selection Considering Selection Intensity, Gain, Diversity, and Productivity

**Figure 1:**
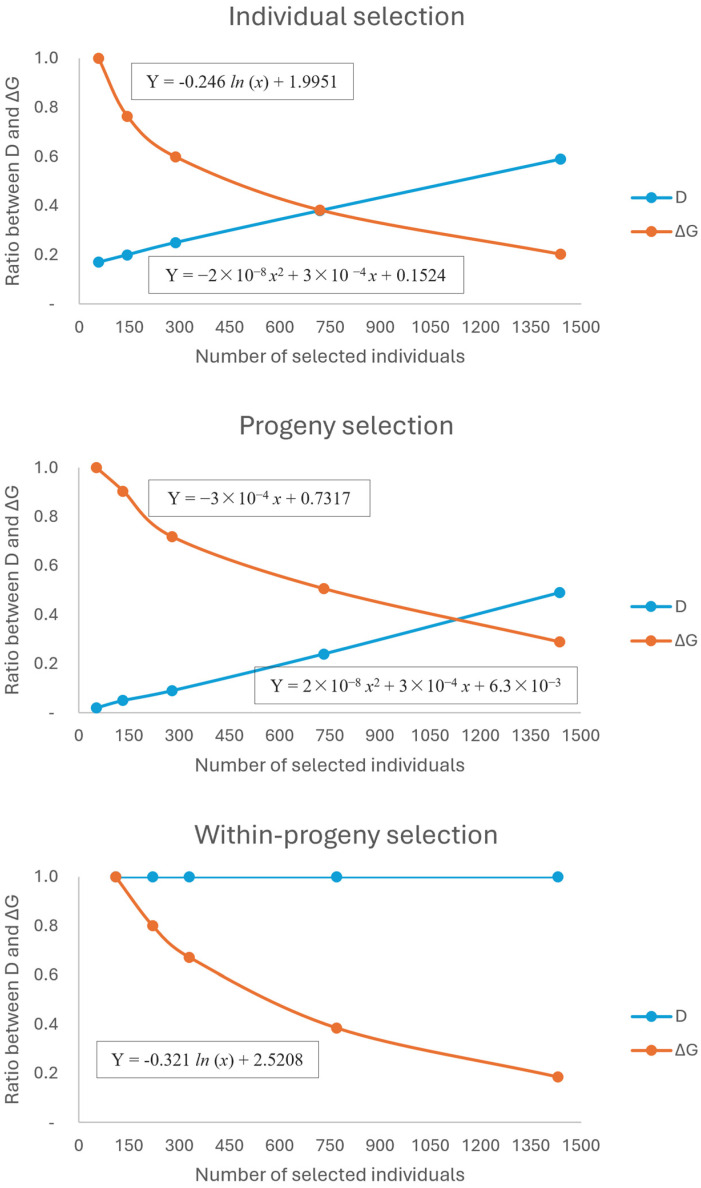
Optimal point maximizing the genetic gain (ΔG) and genetic diversity (D) for different selection methods in *Araucaria angustifolia* provenance and progeny test in Brazil.

The expected genetic gains varied from 10.2%, with 1430 individuals using within-progeny selection, to 124.4%, with 58 individuals in the individual selection (Figure 1). As expected, the within progenies method maintains the highest genetic diversity regardless of the selection intensity. For the individual selection method, diversity ranged from 0.17 (58 individuals) to 0.59 (1438 individuals). For the progeny selection method, this varied from 0.02 (52 individuals) to 0.49 (1437 individuals).

For individual selection, the optimal point between D and ΔG was achieved with a selection of 750 individuals, ensuring 45.6% of genetic gain and 0.37 of diversity (Table 1). For progeny selection, although the diversity (0.38) was higher than that obtained by individual selection (0.37), the number of selected individuals was higher (1164) and the genetic gain was lower (28.6%). Using the within-progeny selection method, both the diversity (1) and genetic gain (54.9%) were superior to other methods. Also, the number of individuals selected was the lowest (110).

Regarding productivity, the total wood volume (TWV), which is the sum of the thinned volume at 33 years (WV_33_) and the volume of the remaining trees at 40 years (FWV_40_), showed better performance in the optimized progeny selection scenario compared to individual selection and within-progeny selection (Table 1). This is because, despite the lower volume of wood harvested at 33 years, the greater number of remaining trees increased the mean annual increment (MAI), resulting in a higher final volume at the 40-year harvest.

### 2.2. Economic Evaluation and Sensitivity Analysis of Production Scenarios

Two production alternatives were proposed to be simulated across the three optimized scenarios indicated in Table 2: wood and wood + nuts. Thus, six new scenarios for production and management were established.

These scenarios analyze the acquisition of the experimental area, considering that it is a productive 33-year-old plantation which is thinned (with an intensity according to the selection method) and whose remaining trees are conserved for seven years until the final wood harvest. In the case of the wood production alternative, the purpose is exclusively to conserve the genetic material in a selected population during this period to establish the next breeding generation and commercial planting. Meanwhile, the wood + nuts production alternative would also allow for the generation of seeds with a certain degree of improvement.

#### 2.2.1. Wood Production

The scenarios were analyzed with a minimum acceptable rate of return (MARR) of 8%. The progeny selection scenario showed the highest total revenue (TR), total costs (TC), and total net revenue (TNR) (Table 2). On the other hand, the within-progeny selection scenario provided the highest net present value (NPV) and benefit–cost ratio (B/C), followed by the individual selection and progeny selection methods.

**Figure 2:**
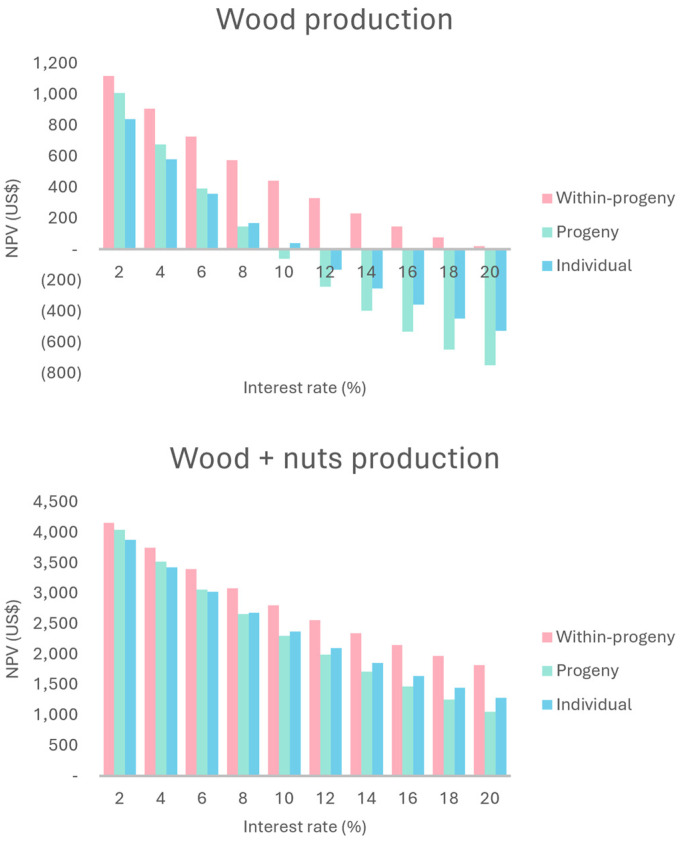
Tendency of the net present value (NPV) for wood and for wood plus nut production scenarios due to different attractiveness rates in a provenance and progeny test of *Araucaria angustifolia* at 33 years old in Brazil.

In the sensitivity analysis, when the MARR is reduced to 2%, there is an inversion in the profitability order between the progeny selection and individual selection scenarios (Figure 2). Additionally, the difference between the three selection scenarios decreases, due to the characteristics of each cash flow and the effect of the MARR on them. Conversely, a MARR of 10% makes the progeny selection option unviable. At 12%, only the within-progeny selection option remains viable, proving to be the best scenario throughout the entire MARR variation (2–20%).

**Figure 3:**
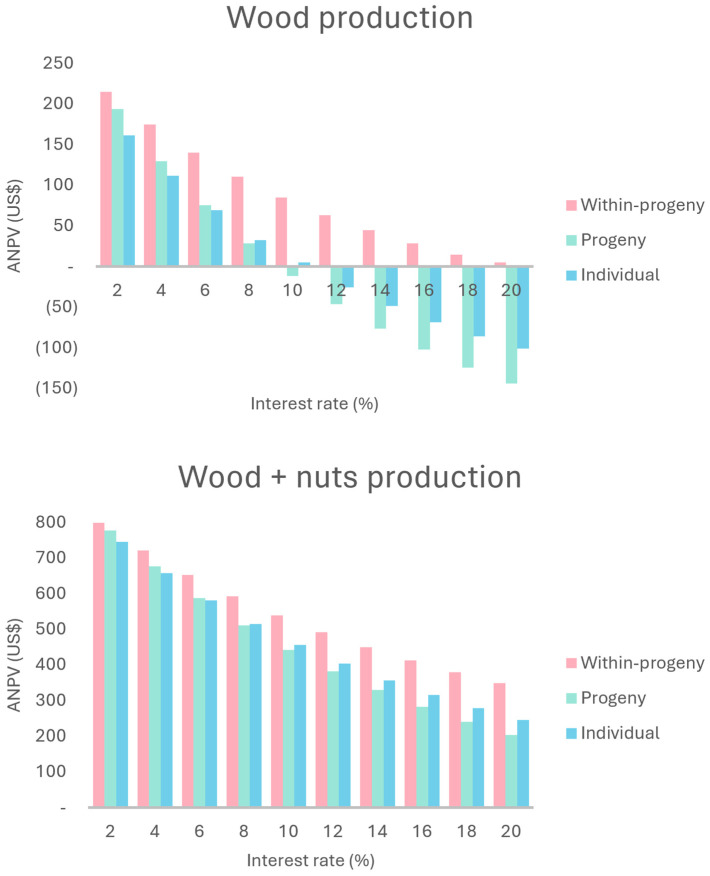
Tendency of the annualized net present value (ANPV) for wood and for wood plus nut production scenarios due to the different attractiveness rates in a provenance and progeny test of *Araucaria angustifolia* at 33 years old in Brazil.

**Figure 4:**
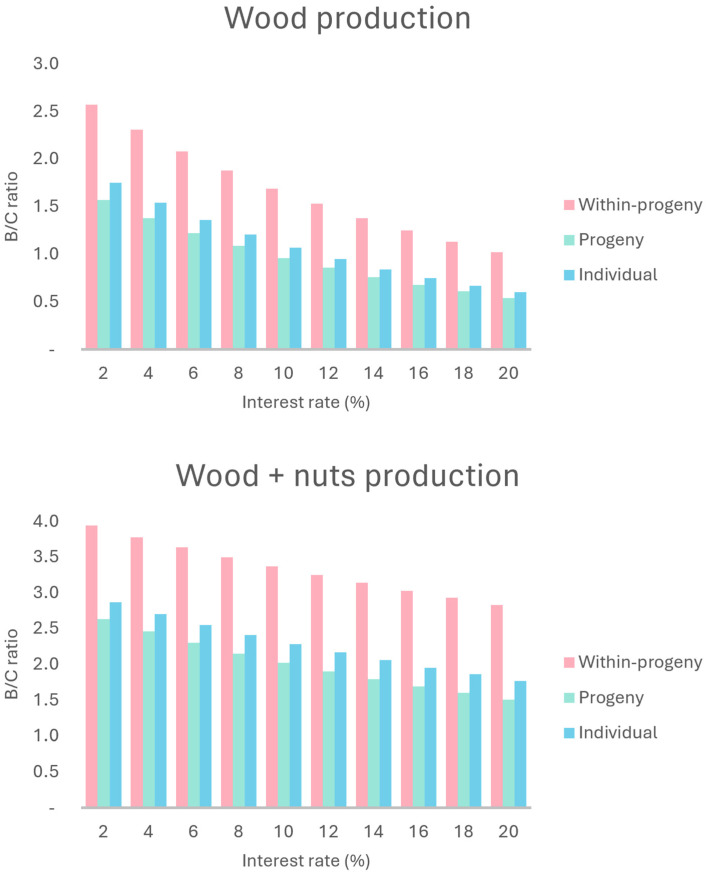
Tendency of the benefit–cost (B/C) for wood and wood plus nut production scenarios due to different attractiveness rates in a provenance and progeny test of *Araucaria angustifolia* at 33 years old in Brazil.

For annualized net present value (ANPV), the analysis follows the same trend observed for NPV (Figure 3). Sensitivity analysis was also applied to the benefit–cost ratio (Figure 4), where the individual selection method always showed better results than progeny selection (unlike the NPV and ANPV), both of which were surpassed by within-progeny selection.

#### 2.2.2. Wood + Nuts Production

These scenarios considered the use of wood in the final harvest after seven years and the annual production of araucaria nuts for six years. There was no differentiation in nut production between the selection methods.

With a MARR of 8%, the production of araucaria nuts impacted the TR found in wood production, generating an increase of USD 4008.55. As for the TC, there was an increase of USD 751.88 (Table 2). It was assumed that the nut harvest was carried out by third parties. The TC could be minimal if the harvest were conducted by the farmer (owner). For wood production, among the selection types analyzed, the progeny selection showed the highest TR, TC, and TNR values for the same reasons presented earlier.

In the sensitivity analysis, the within-progeny selection scenario returned the best results for the NPV (Figure 2), ANPV (Figure 3), and the benefit–cost ratio (Figure 4). While differences in profitability order between progeny selection and individual selection are still observed depending on the MARR considered, it is evident that, throughout the entire variation (2–20%), all three scenarios are profitable. The internal rate of return (IRR) varied from 37.46 (progeny selection) to 130.75 (within-progeny selection). The economic feasibility analysis using the land expected value (LEV) assumes premises, as discussed earlier. The lowest LEV estimate was for the progeny selection scenario, at USD 6378.86.

## 3. Discussion

### 3.1. Optimal Point between Genetic Gain and Diversity

The intensity of selection affects genetic diversity and genetic gains differently, depending on the selection method adopted. There is an inverse relationship between the intensity of selection and the number of individuals selected and genetic diversity: the higher the selection intensity, the fewer the number of remaining trees in the population and, consequently, the lower the genetic diversity. However, the remaining trees concentrate the highest genetic gain compared to the original population. This effect was observed in all scenarios except for the within-progeny selection method, which retains the total genetic diversity by ensuring the presence of at least one representative from each evaluated progeny. The diameter of a tree depends on the dimensions of its crown, which, in turn, are influenced by the amount of space available for tree growth [6,20]. This relationship is crucial because, despite the lower volume of wood harvested at 33 years, the increased space available to the remaining trees enhanced their mean annual increment (MAI). Consequently, this led to a higher final volume at the 40-year harvest.

Optimization was used as a tool to achieve a balance between genetic diversity and genetic gain. This optimization generated three hypothetical populations, one for each selection method, and the economic evaluation suggests that it is possible to establish new and more productive populations with the selected progenies and individuals for breeding and genetic conservation. Although the individual selection method provides considerable genetic gain, the diversity was reduced to nearly one-third of the original, and the number of individuals required to maintain the population in this scenario is too high for a future productive area [21,22]. The optimal point for progeny selection shows that this method is also not the best for achieving the proposed objectives. In general, the test indicates the need for thinning. Therefore, the number of trees selected to remain in the population should be low [22]. To advance in breeding generations, a population with an effective size of around 50 individuals is sufficient [23]. The optimal point considering the within-progeny selection method presents the best results for the objectives of this study.

Selective thinning based on this proposal will allow for the establishment of a seed orchard with high productivity and high genetic diversity. Having a seed-producing area for an endangered species is beneficial. However, the ability to provide material with tested genetic variability is a notable aspect of good management practice, helping to mitigate the degradation of araucaria forests [6]. It is important to note that the species studied is dioecious, so it is recommended to maintain the same number of male and female plants for each family [24]. This would require doubling the number of selected individuals to 220, which would result in a reduction in genetic gain to 44%. The experimental area is 2 hectares, leaving 110 individuals per hectare.

The trees removed during thinning at age 33 belonged only to the classes with the lowest DBH, in all three scenarios. At the final cut, at age 40, the DBH classes expanded to larger diameters, resulting in better-quality wood, as an expected beneficial effect of the thinning performed.

### 3.2. Economic Evaluation and Sensitivity Analysis of Wood Production Scenarios

In the economic analyses, at first, progeny selection shows higher TR and TNR. This occurs because of a higher number of remaining trees, which generates more wood volume and trees with bigger diameters. Consequently, it provides wood of higher prices in the final cut, reflecting on projected revenue. On the other hand, the cost of the forest (TC) was higher due to the higher number of remaining trees [25]. The NPV for the progeny selection scenario was not higher than the other scenarios due to its worse TR/TC ratio (1.77). The NPV and ANPV were higher than zero for all scenarios. The analyzed scenarios are viable, with the within-progeny selection being the most appealing. The projects would not be economically viable if the NPVs were negative. The higher is the NPV, the more attractive the investment is [26]. Concerning attractiveness, the ANPV can be compared to the opportunity cost of land use. In the region, the lease value ranged from 156.64 to 281.95US$ ha^−1^ month^−1^ for corn or cane crops, respectively [27]. None of scenarios showed an equal or higher NPV, meaning that conservation would be less appealing than alternative uses. The IRR ranged from 9.38 (progeny selection) to 20.44 (within-progeny). A project is economically viable when the IRR is higher than the minimum acceptable rate of return (MARR) [28]. The MARR adopted for this study was 8% per year. As all alternatives resulted in an IRR higher than 8%, none would be discarded.

The LEV varied widely between scenarios. According to Oliveira [29], a project is only viable if the LEV is greater than the land acquisition cost (LAC). In the region of Itapeva, SP, the mean price for pasture or reforestation is USD 4229.32 per hectare [30]. Therefore, none of scenarios would be viable by this criterion. However, the LEV is an appropriate indicator for projects whose production cycles are perpetuated, allowing the comparison of alternative management regimes with different rotation lengths [31]. That would not be the case for the analyzed scenarios. The seven years of wood production cycle considers a growth between 33 years and 40 years to be inappropriate. Machado and Bacha [32], based on experimental information in the same region, analyzed several simulated scenarios for araucaria wood production with 25-year production cycles and observed economic viability for the indicator when the MARR was less than 10%. The NPV is the most consistent from the economic evaluation indicators, but it also has shortcomings and limitations. The NPV and the B/C are insensitive to the project duration. On the other hand, these are sensitive to changes in the MARR [33].

The benefit–cost ratio was higher than one for all scenarios (B/C > 1), which means that all scenarios can be considered viable [34]. This indicator is widely used because it is relatively easy to interpret compared to other economic viability indicators. Therefore, all proposed scenarios would be approved because these are above this reference value. Finally, the economic payback (EPB) is at seven years once the first and only revenue comes from the final cut. Due to the inadequate experiment management and the inappropriate location for araucaria growth, the proposed conservation could be impracticable. However, the economic parameters indicate the viability of conservation through use, considering the harvest of wood at the end of the proposed cycle. The sensitivity analysis concerning the B/C, when compared to the NPV, shows different behavior due to the change in the MARR [35]. Despite the behavior of the NPV and ANPV being very similar to that of the MARR, it is observed that the reduction in the MARR did not mitigate the difference between the scenarios. The inversion in order of preference observed when the MARR was 2% also did not occur.

### 3.3. Economic Evaluation and Sensitivity Analysis of Wood and Nuts Production Scenarios

The araucaria nut production effect on the B/C, NPV, and ANPV was positive. When the B/C rate is higher than 1, the project is viable [36]. In this case, the lowest estimate was 2.15, being all scenarios that are viable. Regarding the selection of projects, the B/C method is consistent with the NPV and ANPV. So, the projects have the same evaluation regarding rejection or not [37]. Since all alternatives demonstrate an IRR higher than the MARR of 8%, none of them would be discarded. The analyzed scenarios are viable because the estimates of the NPV and ANPV are higher than zero, with the within-progeny scenario of selection being the most attractive. Attractiveness could be compared to the opportunity costs of land use (156.64 to 281.95US$ ha^−1^ month^−1^ for corn or cane crops, respectively). The ANPV of the three scenarios was higher than this value, showing that conservation became more attractive to USD 4229.32 per hectare. Therefore, even if the production cycle conditions are not adequate, the inclusion of the annual nuts production assured an important long-term index when considering forest project implantation [38].

The production of araucaria nuts for the remaining trees after selective thinning, added to the wood production after seven years, was highly positive. The economic evaluation parameters for all proposed scenarios signaled, besides the economic viability, the improvement of these indexes. Also, with the LEV, the effect of nut production on long-term viability became clear. In this condition, the option within progenies is the most attractive considering the NPV parameter. However, this parameter is sensitive to changes in the MARR [39]. In the sensitivity analysis for wood production, MARRs at 10% and 12% would make progeny and individual selection options impracticable, respectively. For the wood and araucaria nuts production, a MARR higher than 8% intensifies the differences between the options. However, it does not make any of them impracticable until the maximum MARR is considered at 20%. Sensitivity analysis can also be applied to other indicators of economic evaluation. The ANPV follows the same trend observed for the NPV. The analysis of the MARR changing behavior is also the same. Like the NPV, the B/C is also sensitive to MARR changes [40]. However, the high B/C rates were not affected by any MARR value (2% to 20%). None of the assumed MARRs render one of the three scenarios impracticable or even change the profitability order.

Throughout this study, we sought to answer the question of whether genetic conservation of trees in forest plantations (in this case, Paraná pine) can be economically viable through use of forest subproducts (in this case, nuts). Our results provide a satisfactory answer to this guiding question. Moreover, we believe it is important to highlight that these satisfactory results were obtained while maintaining the genetic variation in the selected populations. This is not a minor point, considering that genetic variation plays a crucial role in enhancing the adaptability of species to changing climatic conditions and emerging diseases while also contributing to increased productivity, making it a key factor in ensuring the resilience and sustainability of forest ecosystems [9,41].

Despite our efforts to review the literature for similar studies addressing the conservation of endangered species through their use, particularly those focused on ex situ conservation strategies (in our case, plantations designed as a provenance and progeny test with the aim of developing a genetically improved seed orchard), and evaluating not only the economic viability of conservation through use but also the simultaneous maintenance of genetic diversity within populations, we found no comparable studies to discuss or contrast our results with. Nevertheless, we did identify studies that assess conservation efforts for threatened species’ genetic resources with promising and positive outcomes, such as the work of Herrmann [42] on Pehuén (*A. araucana*) in the Chilean Patagonia and Kainer et al. [43] on the Amazona nut (*Bertholletia excelsa*) in the Brazilian Amazon, among others. However, these studies lack in-depth evaluations of the economic and genetic sustainability of their initiatives, a gap we aim to address. We hope that the results and conclusions presented in this research will serve as a guide for evaluating similar initiatives in other species, or even the same species in different regions. Furthermore, we envision this work as a foundation for more rigorous future studies, incorporating enhanced economic parameters and indicators, such as the price per kilogram of pine nuts or the number of nuts produced per tree, to refine the approach we have initiated here.

## 4. Materials and Methods

### 4.1. Provenance and Progeny Test

The trial was established at the Itapeva Experimental Station, located at 24°17′ S, 48°54′ W, and at an elevation of 930 m, in Southeast Brazil. The local climate is tropical, with dry winters and rainy summers. The average annual temperature is 18.6 °C, and the average annual rainfall is 1300 mm [44]. According to Rossi [45], the soil at the site is an association between alic typical dystrophic red oxisol with a medium texture and alic typical dystrophic red oxisol with aclay texture, both with wavy and slightly wavy relief.

A complete block design was adopted using compact families with three replications and five provenances (plots), with 14 to 26 progenies per provenance (subplots), 10 plants per subplot, and spacing of 3 m × 2 m. Two border rows of the same species were planted around the entire experiment to reduce the edge effect on the treatments [44]. The soil was prepared by plowing and harrowing. In the first year, ant control, mechanical mowing, and 1% replanting of seedlings were performed. From the second to the fifth year, ant control, mowing, and harrowing were carried out. From the sixth year onward, management was limited to harrowing and mowing once a year. Survival (SUV, %), plant height (H, m), and diameter at breast height (DBH, cm) were measured at age 33 years. Individual volume (VOL, m^3^) was calculated using the following equation:

(1) $$VOL=\frac{{\left(DBH\right)}^{2}}{40,000}\times FF\times H$$

with FF being the form factor (0.6) given by Sanquetta et al. [46].

### 4.2. Simulation of Selection Strategies

**Figure 5:**
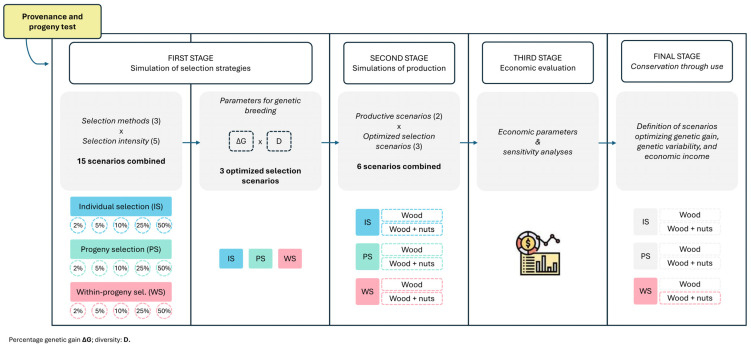
Workflow for evaluating selection strategies and production scenarios with economic and sensitivity analyses for optimal conservation and use. First Stage: Testing scenarios to choose the most suitable selection strategy considering the simultaneous maximization of genetic gain (ΔG) and diversity (D). Second Stage: Testing productivity scenarios based on the optimized selection strategies from the first stage. Third Stage: Economic evaluation and sensitivity analysis of the productivity scenarios tested in the second stage. Final Stage: Selecting the best scenario based on the results obtained in the previous stages, aimed at identifying the most productive scenario worth conserving for its intended use (conservation through use).

Fifteen selection scenarios for volume were simulated to establish a breeding population that considers the best combination of genetic gain and genetic diversity conservation. The genetic values for volume were estimated in Machado et al. [41]. The fifteen scenarios combined three selection methods, individual selection (IS), progeny selection (PS), and within-progeny selection (WS), with five selection intensities (2%, 5%, 10%, 25%, and 50%) (Figure 5). For all these scenarios, a 1:1 sex ratio was considered, as it has been verified that, in natural populations, this ratio does not significantly diverge from one (1:1) [47,48,49].

Once the fifteen populations were simulated, the genetic diversity (D) and genetic gain (ΔG, %) were estimated for each, according to Resende [18]. Genetic diversity was estimated as:

(2) $$D=\frac{N_{ef}}{N_{fo}}$$

where 0 < D ≤ 1, with N_ef_ being the effective number of selected progenies, which is given by:

(3) $$N_{ef}=\frac{{{(\Sigma N}_{f})}^{2}}{{{\Sigma N}_{f}}^{2}}$$

where k_f_ is the number of individuals selected per progeny and N_fo_ is the original number of progenies. To determine the intensity of selection that maximizes both D and ΔG, the values were compared using a relative scale of 0.0–1.0. Quadratic equations were used to estimate the regression curves for D and ΔG [50]. The point of intersection of the curves was used to determine the optimal selection intensity for each method.

The three simulated breeding populations were subjected to additional simulations of wood harvest productivity and nuts production, as well as the evaluation of economic parameters estimated from the exploitation of these breeding populations.

### 4.3. Simulations of Wood and Nuts Production

Two production alternatives were proposed: (1) wood and (2) wood + nuts. These were simulated for the three breeding populations defined in the previous step, resulting in six new scenarios (Figure 5). The increase in wood volume was considered between 33 and 40 years, starting from the populations already thinned according to each selection method. The simulation for each of the six scenarios was carried out using the EMBRAPA Florestas’ SISAraucaria software (version 2003) [29]. To define the forest acquisition volume at 33 years (FAV_33_), equivalent to the initial volume for a new economic cycle, the final wood volume harvested at 33 years (FWV_33_) and the final wood volume harvested at 40 years (FWV_40_) were simulated, the latter considering thinning (harvested wood volume) at 33 years (WV_33_), with the thinning intensity corresponding to each selection method. The forest acquisition volume at 33 was then defined as:

(4) $${FAV}_{33}={FWV}_{33}-{WV}_{33}$$

In both cases, the multiple uses of the wood were considered according to Oliveira [29].

Araucaria nut production varies widely with the soil conditions, genetic background, local climate, etc. For this study, we adopted the productivity specifications of Figueredo Filho et al. [51] which indicate a production of 10 araucaria cones per tree and an average weight of 376 g seeds per cone.

Araucaria nuts production was estimated based on the area capacity regardless of the number of trees remaining for each scenario considered. Then, nut production (PROD_nut_) was calculated as follows:

(5) $${PROD}_{nut}=N_{plants}\times Q_{pine}\times {Pe}_{nut}$$

where N_plants_ is the number of female trees, Q_pine_ is the amount of araucaria cones per tree, and Pe_nut_ is the weight of seeds per cone, in Kg. The support capacity for the area was evaluated at 180 female trees per hectare. The male/female ratio is 1:1, implying that PROD_nut_ = 180 × 10 × 0.376. Therefore, nut production 677 kg/ha.

### 4.4. Economic Evaluation

Simulations of economic parameters were performed using Planin Software (version 1995) for forest production [29]. The implementation cost was defined as the cost of forest acquisition (CFA) at 33 years old, since it had already been implemented. This cost was calculated as:

(6) $$CFA=\Sigma_{i}^{n}{IV}_{i}\times P_{i}$$

where IV_i_ is the initial volume of product i in m^3^ ha^−1^, P_i_ is the price of product i in US$, and i is the product type. The wood revenue (WR) from the final harvest was estimated as:

(7) $$WR=\Sigma_{i}^{n}{FV}_{i}\times P_{i}$$

where FV_i_ is the final volume of product i in m^3^ ha ^−1^ and P_i_ is the price of product i in US$. Price information for forest products was obtained from the Department of Rural Economy (DERAL) survey, referencing the 2017 report [52]. Prices for products with a diameter less than 25 cm were estimated based on exotic pine prices. The annual nut revenue (NR) was estimated as:

(8) $$NR=Q_{p}\times P_{p}$$

where Q_p_ is the nut production per hectare and P_p_ is the nut price per kg in US$. The average price received by producers for nuts in producing regions was 0.99 US$/kg in 2017 [52].

Sensitivity analysis considered different rates of attractiveness. We first estimated the net present value (NPV) as:

(9) $${NPV}_{i}=\Sigma_{n=0}^{t}F_{n}{\left(1+i\right)}^{-n}$$

where the NPV_j_ is the net present value of a financial flow of the j alternative, t is the time from the cash flow, n is the total number of periods (years), F_n_ is the net cash flow, and i is the discount rate. The annualized net present value (ANPV) is the transformation of the NPV of a financial flow at the minimum acceptable rate of return (MARR), represented in this equation by the term i into a uniform annual series, which is equivalent from its multiplication by the term:

(10) $$\frac{i{\left(1+i\right)}^{t}}{{\left(1+i\right)}^{t}-1}$$

At the end of each period (year), there is a uniform portion whose sum of the discounted values results in the NPV of the financial flow [29]. The internal rate of return (IRR, i*) is given by the following equation:

(11) $$\Sigma_{n=0}^{t}F_{n}{\left(1+i^{*}\right)}^{-n}=1$$

where the minimum acceptable rate of return (i) adopted was of 8% per year. The benefit–cost ratio (B/C) of a project indicates how many units of capital received with benefits (B) are obtained for each unit of invested capital (C), and it was estimated as:

(12) $$B/C=\frac{\Sigma_{n=0}^{t}R_{n}{\left(1+i\right)}^{-n}}{\Sigma_{n=0}^{t}C_{n}{\left(1+i\right)}^{-n}}$$

where R_n_ is the revenues and C_n_ the costs [29]. The economic viability of reforestation projects can also be evaluated with the Faustmann equation [53], also known as land expectation value (LEV), following the equation:

(13) $$LEV=\frac{\Sigma_{n=0}^{t}(R_{n}-C_{n}){\left(1+i\right)}^{t-n}}{\left({\left(1+i\right)}^{t}-1\right)}$$

The “payback” is a method that calculates the number of years for the company to recover the capital invested in the project. The economic payback (EPB) occurs when the following relationship is satisfied:

(14) $$EPB=k,such\;that\;\Sigma_{i=0}^{k}\left[\frac{F_{i}}{{\left(1+j\right)}^{i}}\right]\geq 0$$

with F_i_ being the cash flow and j being the minimum acceptable rate of return (MARR) [54]. In this study, the value of 8% was adopted as the MARR. For the sensitivity analysis, the interest rate varied from 2 to 20%.

## 5. Conclusions

The high genetic control of the growth traits permits the genetic gains for further generations of productive forests. For this studied area, considering productive and conservation purposes, the ideal would be to select the best male and female individuals of each progeny through the harvesting of the non-selected trees (the within-progeny selection method). If maintaining only these individuals, the area can be converted into a nuts production forest. This new seed orchard would be a seed source of high genetic quality for small and medium farmers interested in the species cultivation for wood and nut production.

The parameters indicated the economic viability of the proposed productive forests. They show that the investment can be more profitable and safer with araucaria nut production alongside the wood. The return rates proved to be attractive for investment, even considering pessimistic scenarios on the sensibility analysis. Therefore, the conservation through use proved to be a valid tool in protecting the species from extinction and promoting local socio-economic development. However, government agency incentives, such as tax breaks, would be necessary to make this type of investment more attractive.

Araucaria is one of the most studied species in Brazil, primarily in relation to the conservation of genetic diversity and ecological benefits [3,6,9,13,24,41]. However, the connection between economic and genetic aspects has been scarcely studied (or is non-existent). The conservation of this species, both in situ and ex situ, has been carried out in its main natural occurrence regions (South and Southeast Brazil). Several efforts have been made by researchers, companies, and producers to ensure its genetic conservation and sustainable use. Often, all farmers, including women, participate in these research projects. Similar examples of species that could be indicated for the bioeconomy in other biomes include baru nut (*Dipteryx alata*), Amazona nut (*Bertholletia excelsa*), palm trees (such as açaí (*Euterpe edulis*), babassu (*Attalea speciosa*)), yerba mate (*Ilex paraguariensis*), among many others. Therefore, the results of this study could also serve as a model for this and other species of Brazilian flora.

## Figures and Tables

**Table 1:** Optimal number of individuals to be selected in a provenance and progeny test of *Araucaria angustifolia* according to genetic gain, genetic diversity, and selection method in Brazil. Additionally, the wood volume harvested at 33 years of age (WV_33_), final wood volume harvested (FWV_40_), total wood volume (TWV), and mean annual increment (MAI) at 33 and 40 years of age in m^3^.

Selection Method	Number of Individuals	Genetic Gain (%)	Diversity	WV_33_ [MAI]	FWV_40_ [MAI]	TWV
Individual	750	45.6	0.37	58.3 [3.1]	89.5 [3.7]	147.8
progeny	1164	28.6	0.38	42.2 [3.1]	113.4 [3.9]	155.6
Within-progeny	110	54.9	1.00	85.6 [3.1]	49.9 [3.4]	135.5

**Table 2:** Economic parameters for evaluating wood and wood plus nuts production scenarios over 7 years based on an optimal point between genetic gain and diversity for three selection methods in a provenance and progeny test of *Araucaria angustifolia* at 33 years of age.

Parameter	Production System by Optimization Scenario					
	Wood			Wood + Nuts		
	Individual	Progeny	Within-Progeny	Individual	Progeny	Within-Progeny
N_is_	375	582	110	375	582	110
ΔG	45.6	28.6	44.0	45.6	28.6	44.0
D	0.37	0.38	1.00	0.37	0.38	1.00
TR	2545.63	3206.75	2106.57	6554.18	7215.31	6115.12
TC	1399.82	1807.58	738.57	2151.70	2559.46	1490.44
TNR	1145.81	1399.17	1368.00	4402.48	4655.84	4624.67
NPV	169.66	147.65	574.72	2678.86	2656.85	3083.93
ANPV	32.59	28.36	110.39	514.54	510.31	592.34
B/C	1.13	1.09	1.88	2.41	2.15	3.50
LEV	407.34	354.50	1379.86	6431.69	6378.86	7404.22
IRR	10.09	9.38	20.44	49.74	37.46	130.75
EPB	7	7	7	3	4	2

N_is_: number of individuals selected; ΔG: percentage genetic gain (%); D: diversity; TR: total revenue (US$); TC: total cost (US$); TNR: total net revenue (US$); NPV: net present value (US$); ANPV: annualized net present value (US$); B⁄C: benefit–cost ratio; LEV: land expectation value (US$); IRR: internal rate of return (%); EPB: economic payback (years). The estimation of the economic parameters for evaluating each scenario was conducted considering a MARR = 8%.

## Data Availability

The data presented in this study are available on request from the corresponding author. The data are not publicly available due to privacy.
